# Supplementary material for: Super-resolution imaging reveals α-synuclein seeded aggregation in SH-SY5Y cells
Source: Commun Biol. 2021 May 21;4:613. doi: 10.1038/s42003-021-02126-w (PMC8139990; doi:10.1038/s42003-021-02126-w)
Supplement: Supplementary file 2 — Supplementary Information [file 42003_2021_2126_MOESM2_ESM.pdf]

Supplementary Information

Super-resolution imaging reveals α-synuclein seeded aggregation in SH-SY5Y cells

Jason C. Sang[1,2], Eric Hidari[1,2], Georg Meisl[1], Rohan T. Ranasinghe[1], Maria Grazia Spillantini[3], David Klenerman[1,2,*]

[1]Department of Chemistry, University of Cambridge, Lensfield Road, Cambridge CB2 1EW, United Kingdom

[2]UK Dementia Research Institute at Cambridge, Cambridge CB2 0XY, United Kingdom

[3]Department of Clinical Neuroscience, University of Cambridge, Cambridge CB2 0XY, United Kingdom

[*]Correspondence to: dk10012@cam.ac.uk

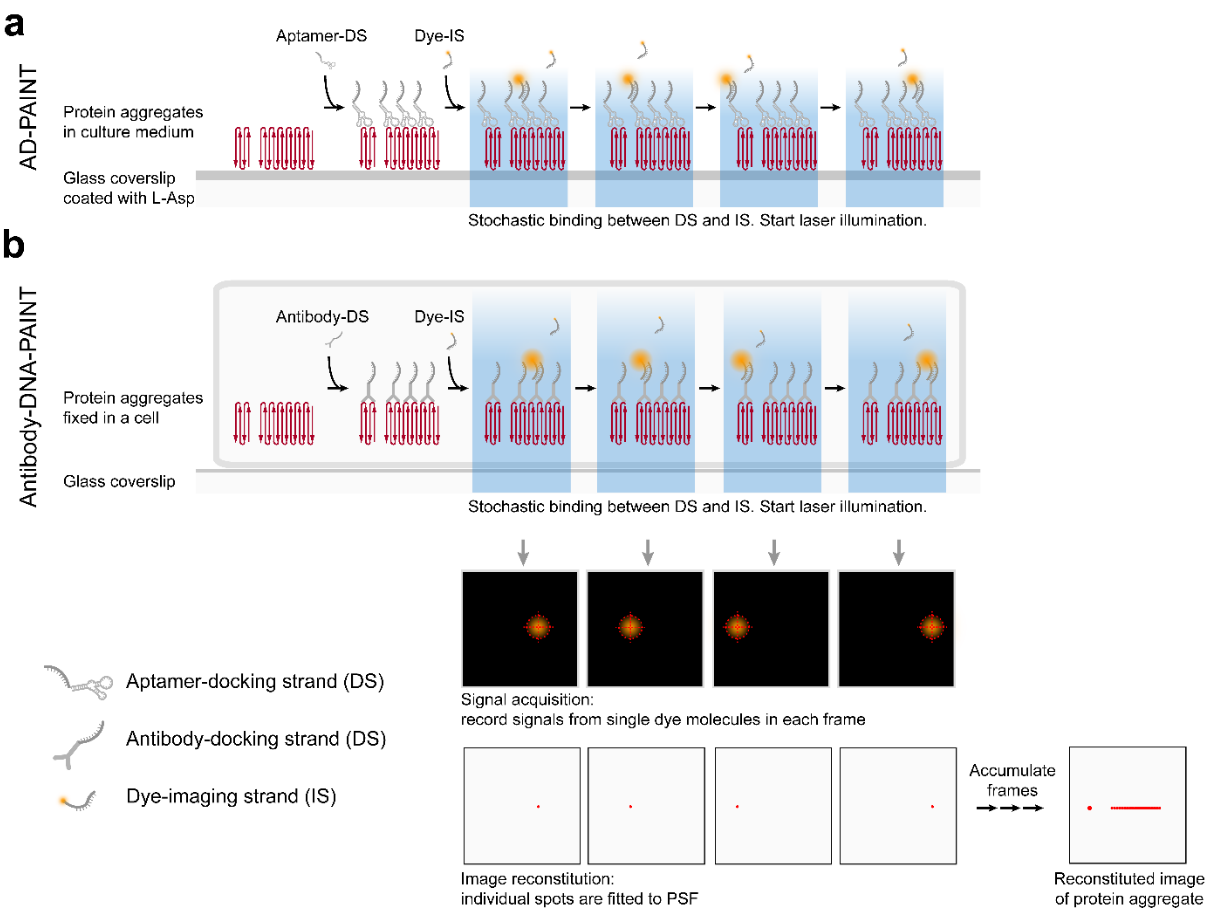

Fig. S1. The principle of AD-PAINT and antibody-DNA-PAINT. The current study utilizes the PAINT (points accumulation for imaging in nanoscale topography) technique, which is a localization-based super-resolution microscopy. Both AD-PAINT (**a**, using aptamers conjugated to a single-stranded DNA, or docking strand, DS) and antibody-DNA-PAINT (**b**, using conformational antibodies conjugated to DS) requires a complementary imaging strand (IS) at a very low concentration, leading to stochastic interaction between DS and IS. Each IS is conjugated to a fluorescence dye. During illumination, the resulting fluorescence is recorded as a function of time, hence generating a series of image frames for further processing. Fluorescence signals detected from a single dye molecule (point source) are blurred by diffraction and can be described by a point spread function (PSF). This phenomenon limits the spatial resolution of conventional microscopies to several hundreds of nanometers. However, if the fluorescence signal is from a single fluorophore then it must be at the center of the fluorescence spot. Therefore, to acquire the exact x-y localization of each molecule, the fluorescence signals are fitted to PSF to determine its center. Then, by accumulating image frames at the same illumination area, more and more fluorescence puncta are recorded over time, and their localizations determined. To correct for any drift, fiducial markers (100 nm-diameter fluorescent beads) are also present in the illumination area. Ultimately, a large number of super-localized events are accumulated, allowing generation of an image with a precision of ~25 nm. Representative raw images, videos, and processed images are shown in Fig. S2 and Supplementary Movies 1-4.

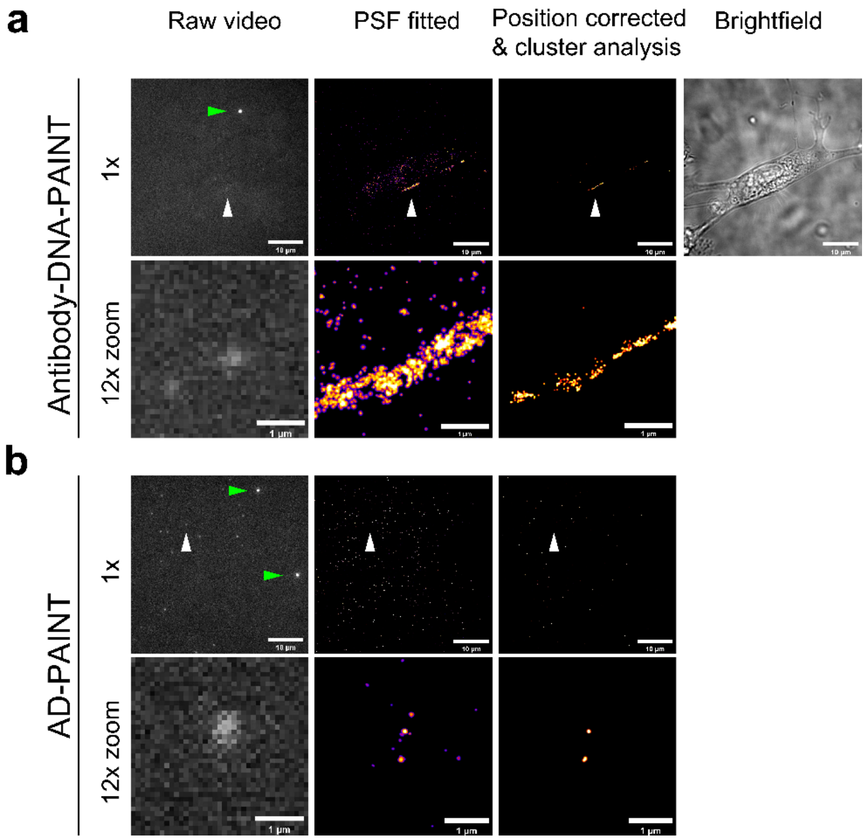

Fig. S2. Raw data and image processing of super-resolution imaging. In antibody-DNA-PAINT (**a**) and AD-PAINT (**b**), fiducial markers (green arrows) are added to provide constant, high-intensity signals to correct for any drift. In raw videos (shown as single frame events here and full videos are available in Supplementary Movies 1-4), individual signals are recorded over time (white arrows). All the signals recorded are then PSF-fitted, followed by cluster analysis (see Methods) for determination of actual aggregates in the image. Antibody-DNA sample is seeded 24h cells and AD-PAINT sample is PFFs. Scale bars for 1x and 12x zoom: 10 and 1 µm.

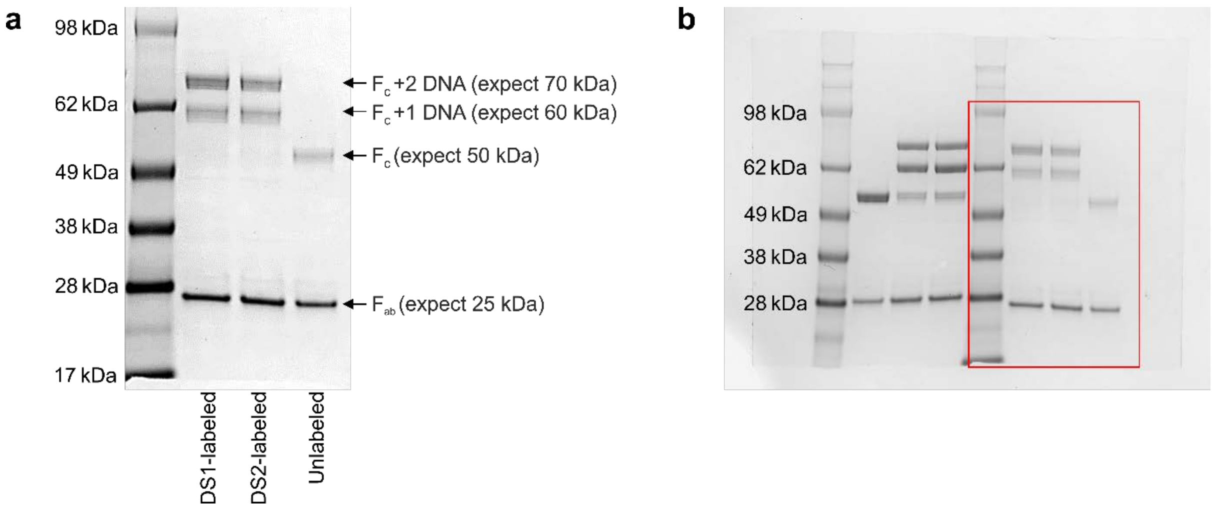

Fig. S3. Characterization of docking-strand-labeled MJFR 14-6-4-2 antibody by PAGE under reducing conditions. (**a**) Cropped image of lanes containing MJFR 14-6-4-2 antibody. (**b**) Uncropped image of whole gel; lanes 2-5 contain a different protein, not used in this study.

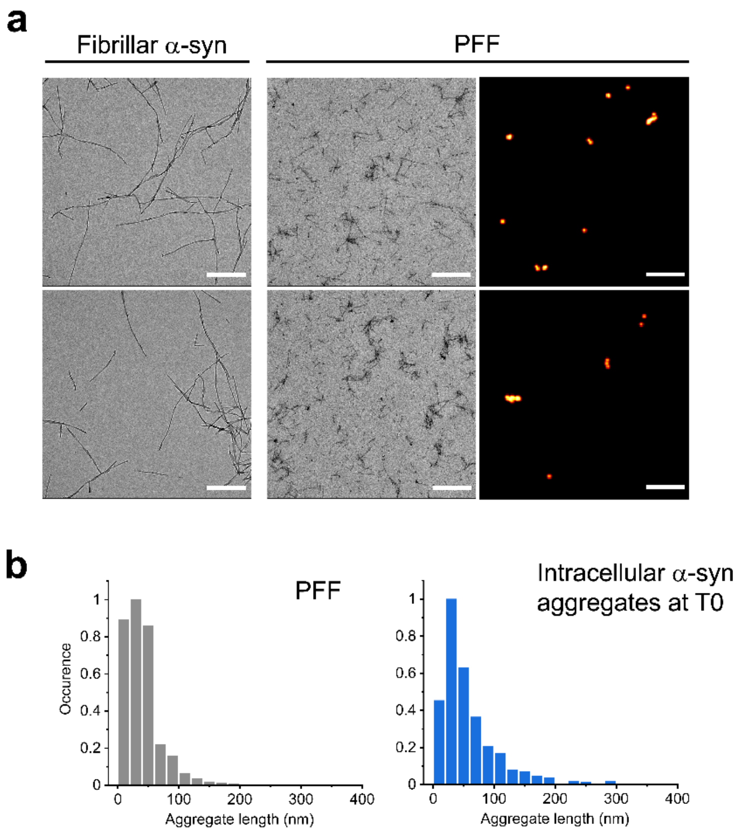

Fig. S4. Representative images of α-syn PFFs and the length distribution. Monomeric α-syn is aggregated at a concentration of 70 μM in PBS/0.1% NaN_3 for 14 days to form fibrils (**a**, left panel) and then sonicated to generate PFFs (**a**, middle panel). Super-resolved images of the PFFs were also shown using AD-PAINT (**a**, right panel, 100× diluted samples than TEM). All scale bars: 500 nm. The length distributions of the PFFs and the intracellular aggregates after seeding (T0) are shown in **b**. Mean values of length for PFFs and intracellular aggregates at T0 are 35 ± 2 nm and 39 ± 8 nm, respectively.

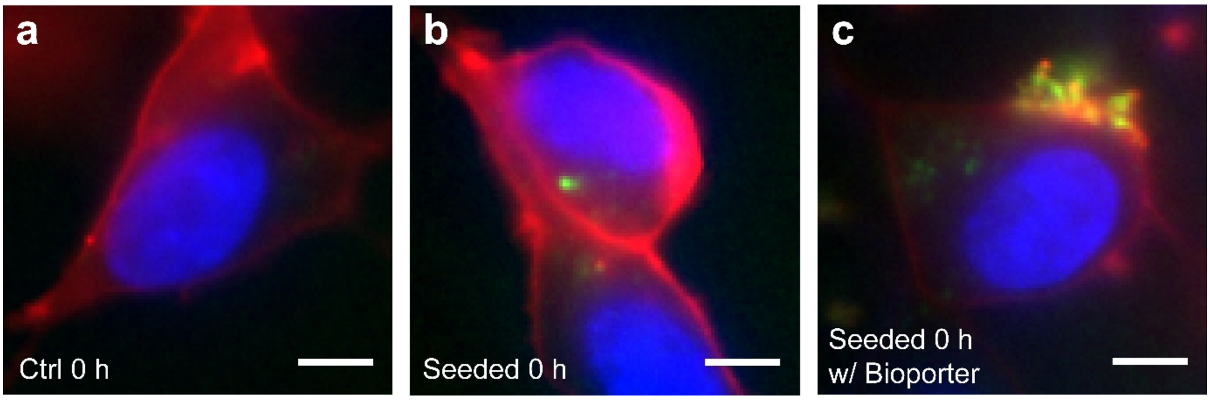

Fig. S5. The uptake of α-syn seeds in SH-SY5Y cells. Cells are incubated either with PBS (**a**, negative cotrol), Alexa 594-labeled seeds alone (**b**), or a mixture of Alexa 594-labeled seeds and Bioporter for 4 h (**c**, time = 0 h). The seeds are at 2.5 μM monomer equivalent. After seeding, cells are imaged in epi-illumination mode. Nuclei are stained blue with Hoechst dye; plasma membranes are stained red with CellMask; Alexa 594-labeled α-syn seeds are shown in green. In the seeded cells in the presence of Bioporter, seeds are observed as green puncta in the cytosol, as well as yellow puncta co-localized with the plasma membrane. Scale bars: 5 μm.

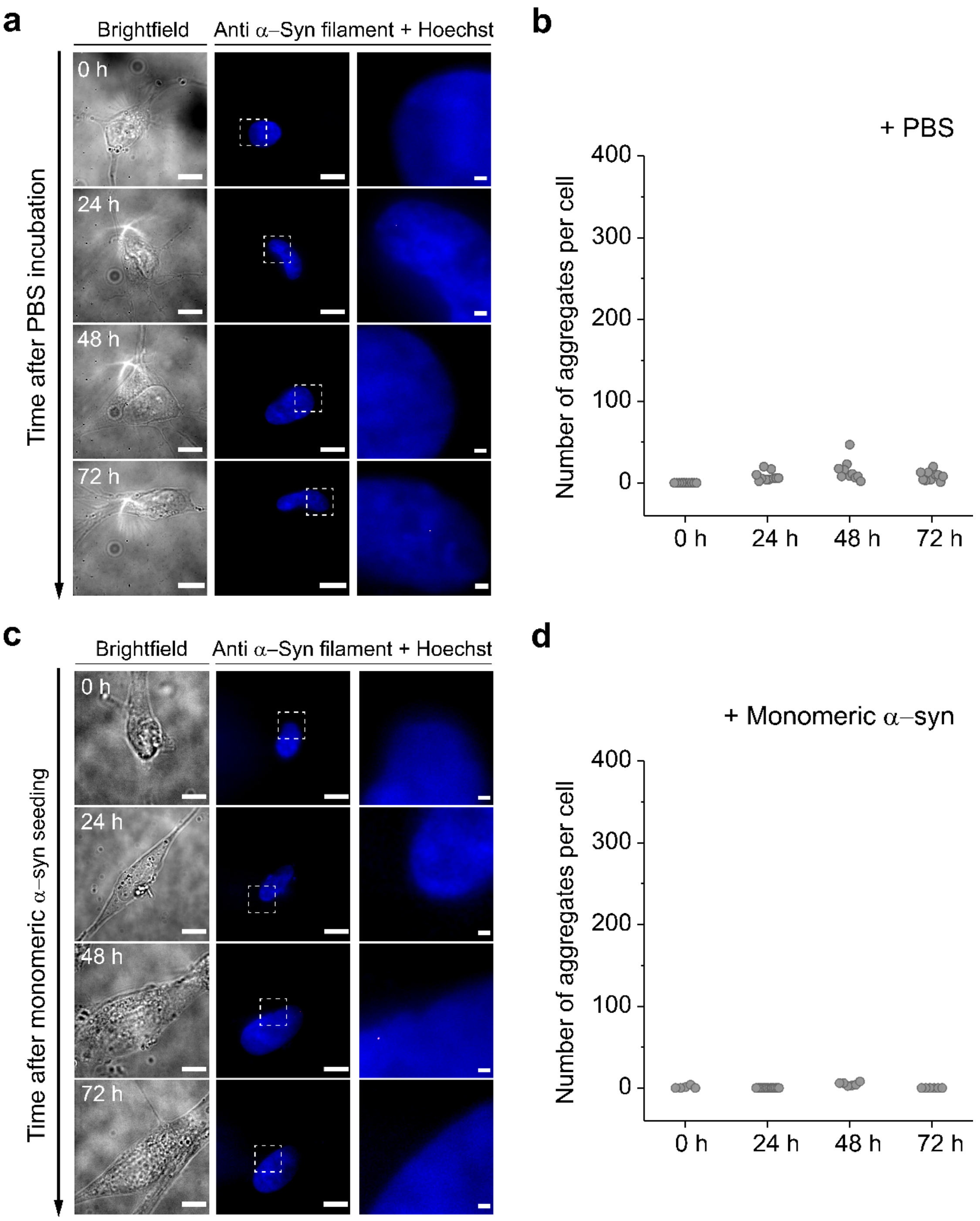

Fig. S6. Incubation with PBS or monomeric α-syn does not induce α-syn aggregation inside cells. SH-SY5Y cells are incubated either with PBS (**a**) or with a mixture of monomeric α-syn (2.5 μM) and Bioporter (**c**) for 4 h, then rinsed with PBS and cultured with fresh DMEM/10% FBS (time = 0 h). Cells at defined time points were immunostained with MJFR-14-6-4-2 and imaged in super-resolution with antibody-DNA-PAINT. Nuclei are stained blue with Hoechst dye; super-resolved α-syn aggregates are shown in red. (**b**) Endogenous α-syn does not self-replicate over time. (**d**) Monomeric α-syn is not efficiently detected by antibody-DNA-PAINT imaging and does not result in α-syn aggregates inside cells. For PBS incubation, *N* (total number of cells imaged) = 10, 10, 10, 10 for 0, 24, 48, 72 h, respectively, from one experiment; for monomeric α-syn seeding, *N* = 5, 14, 6, 6 for 0, 24, 48, 72 h, respectively, from one experiment. Scale bars (from left to right): 10, 10, and 1 μm.

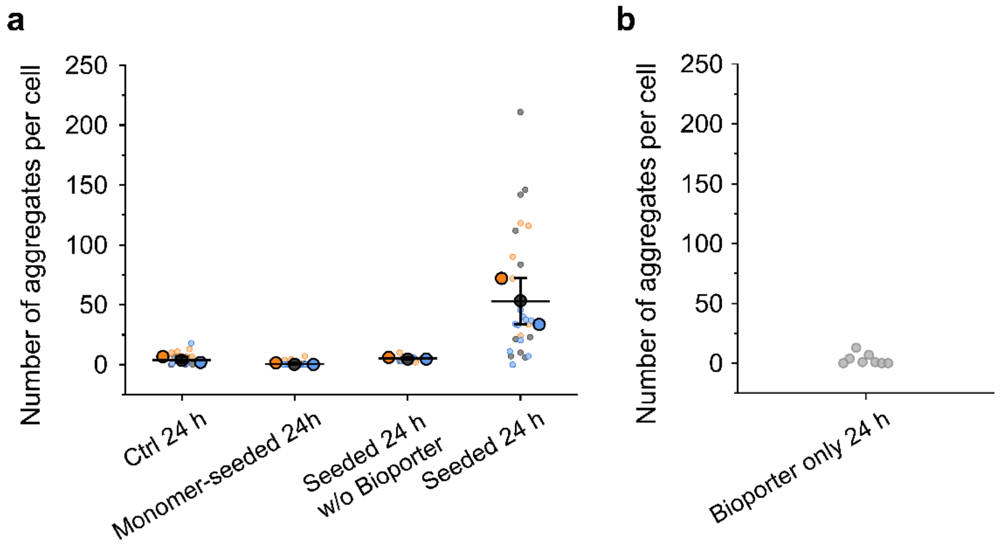

Fig. S7. Bioporter increases seeding probability in SH-SY5Y cells. (**a**) SH-SY5Y cells are incubated with a mixture of seeds (2.5 µM monomer equivalents of α-syn fibrils) and Bioporter for 4 h, then rinsed with PBS and cultured with fresh DMEM/10% FBS (time = 0 h). Cells at defined time points were immunostained with MJFR-14-6-4-2 and imaged in super-resolution with antibody-DNA-PAINT. *N* (total number of cells imaged) = 23, 26, 8, 27 for Ctrl 24 h, Monomer-seeded 24 h, Seeded 24 h w/o Bioporter, Seeded 24 h, respectively. The cells were separately pooled for each biological replicate (n = 3) and the median calculated for each pool; those three medians were then used to calculate the mean (horizontal bars) and standard deviation (error bars). (**b**) Negative control of Bioporter-only group did not show an increase in number of aggregats. Bioporter re-suspended in PBS was incubated with cells in the absence of seeds for 4 h (time = 0 h), then rinsed and cultured for additional 24 h as described above. *N* = 8 from one experiment.

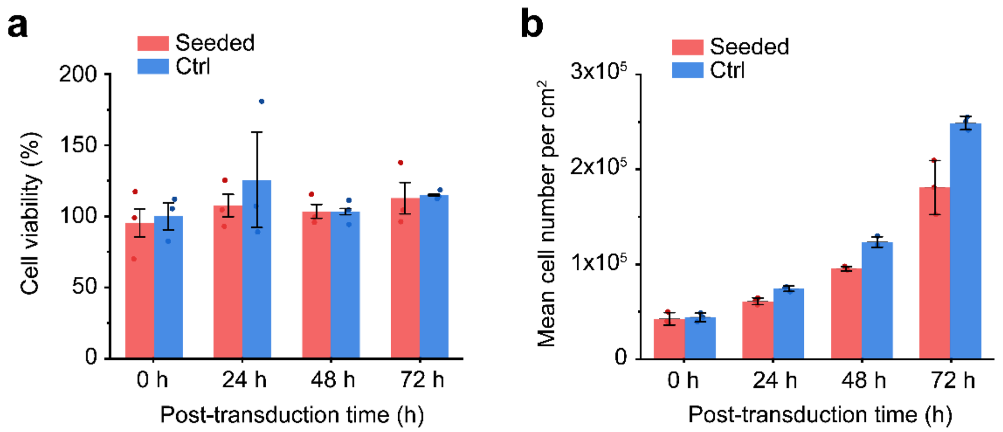

Fig. S8. SH-SY5Y cell viability after seeding. Seeding shows a minor effect on cell viability (**a**) and the dividing rate (**b**) of SH-SY5Y cells. The cells were grown on a glass coverslip in a 6-well plate. The cell viability was measured by lactate dehydrogenase (LDH) assay and no substantial change is found over the 3-day experiment period. The viability of non-seeded control cells (cell density ~5 $\times 10^4$ per cm^2) at time = 0 h was normalized as 100%. Mean ± SD from three biological replicates.

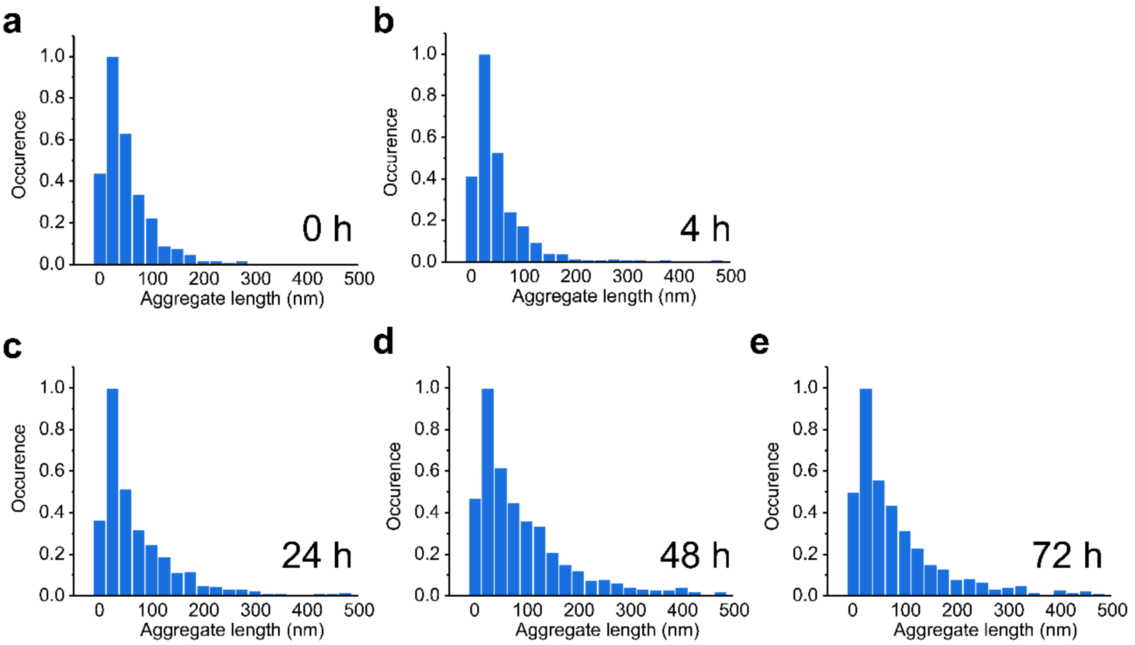

Fig. S9. Length distributions of intracellular α-syn aggregates induced by seeding. The data show the length distribution of all aggregates from Fig. 1.

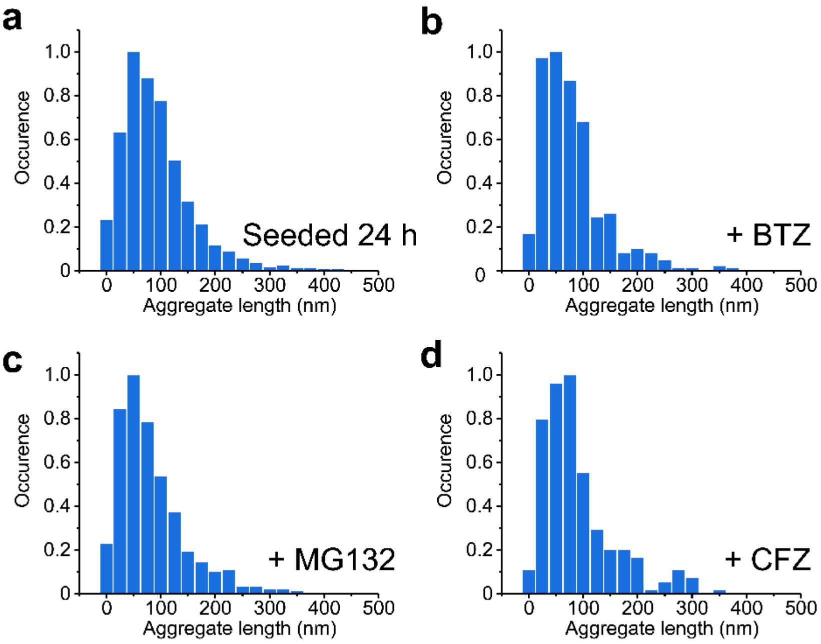

Fig. S10. Length distributions of intracellular α-syn aggregates after proteasomal inhibition. The data show the length distribution of all aggregates from Fig. 2, c and d.

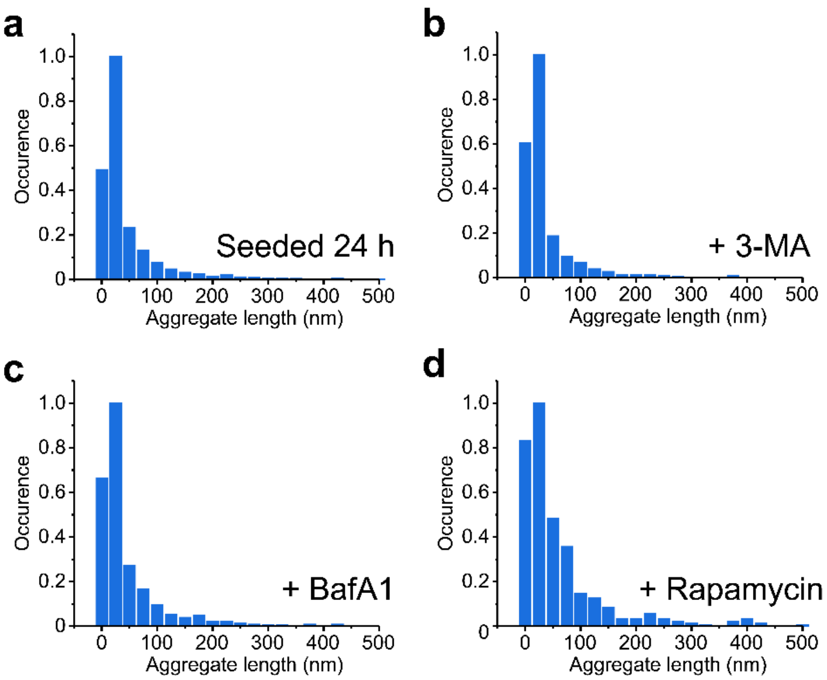

Fig. S11. Length distributions of intracellular α-syn aggregates after treatments of autophagy modulators. The data show the length distribution of all aggregates from Fig. 2, e and f.

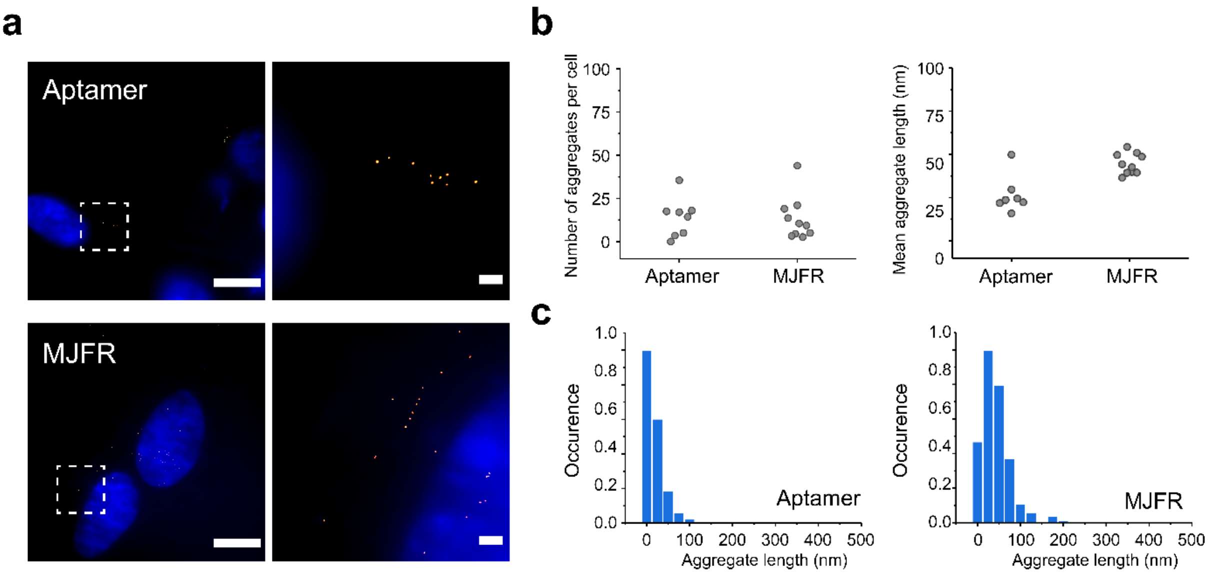

Fig. S12. AD-PAINT imaging with the aptamer gives similar results to antibody-DNA-PAINT. (**a**) SH-SY5Y cells are incubated with a mixture of seeds (final 2.5 µM monomer equivalents of α-syn fibrils) and Bioporter for 4 h, then rinsed with PBS and cultured with fresh DMEM/10% FBS (time = 0 h). Cells at 24 h were imaged in super-resolution either with AD-PAINT or antibody-DNA-PAINT. Nuclei are stained blue with Hoechst dye; super-resolved α-syn aggregates are shown in red. AD-PAINT detects a similar number of aggregates (**b**) with a slightly smaller length distribution of aggregates (**c**) compared with antibody-DNA-PAINT with MJFR 14-6-4-2 antibody. The small difference of ~15 nm in the aggregate size between

the two methods is assigned to the size of the antibody. The data show the length distribution of all aggregates in each experiment. The total number of cells imaged N= 8 and 10 for Aptamer and MJFR, respectively, from one experiment. Scale bars (from left to right): 10 and 1 µm.

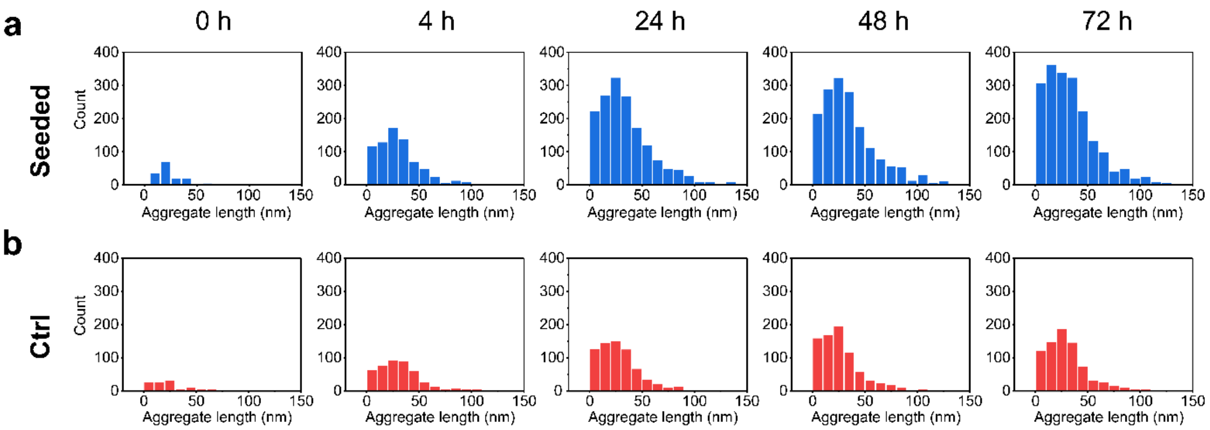

Fig. S13. Length distributions of extracellular α-syn aggregates secreted by seeded and unseeded cells. The data show the length distribution of all aggregates from Fig. 3.

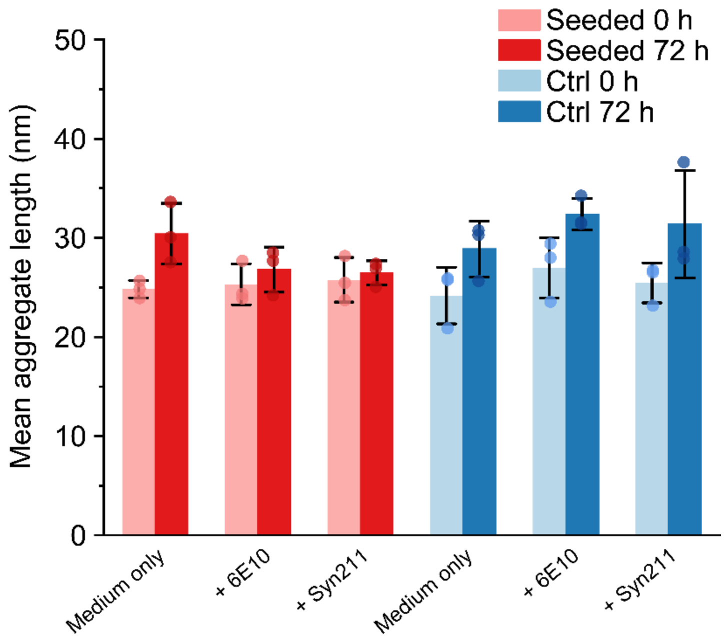

Fig. S14. Increase of the length of secreted aggregates does not have substantial change after immunodepletion. Immunodepletion from culture medium was carried out using 6E10 or Syn211 antibody. The immunodepleted media were then imaged with AD-PAINT. Mean ± SD from three biological replicates.

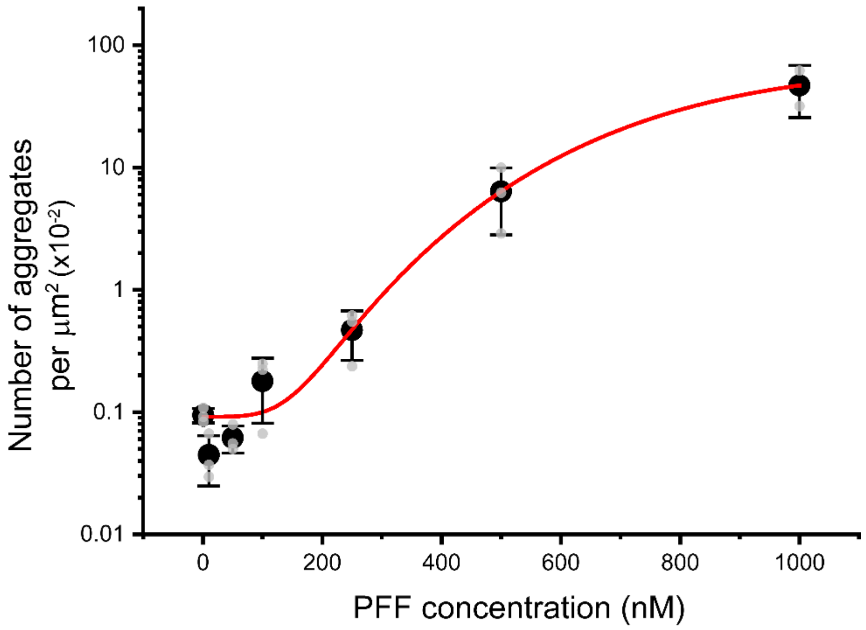

Fig. S15. Calibration of PFFs. α-syn PFFs were imaged with AD-PAINT at defined concentrations. Mean ± SD from three biological replicates. The data are fit to a four-parameter logistic regression. The adjusted R-Square of the fit is > 0.999.

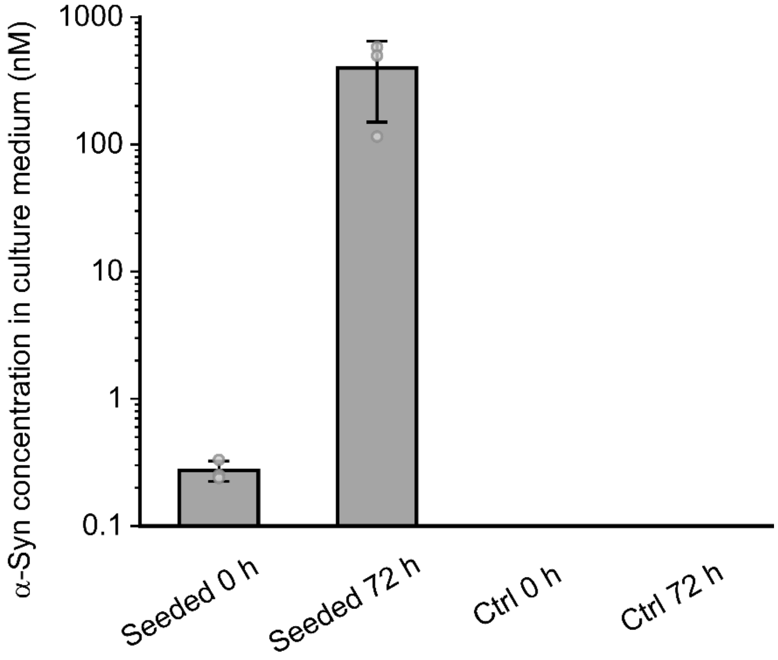

Fig. S16. ELISA quantification of α-syn concentrations in culture medium. Culture medium was collected at defined time points after seeding. Mean ± SD from three biological replicates.

Supplementary references

1. Hoyer, W. *et al.* Dependence of α-Synuclein Aggregate Morphology on Solution Conditions. *J. Mol. Biol.* **322**, 383–393 (2002).

2. Sang, J. C. *et al.* Direct Observation of Murine Prion Protein Replication in Vitro. *J. Am. Chem. Soc.* **140**, 14789–14798 (2018).

3. Jungmann, R. *et al.* Multiplexed 3D cellular super-resolution imaging with DNA-PAINT and Exchange-PAINT. *Nat. Methods* **11**, 313–8 (2014).

4. Whiten, D. R. *et al.* Nanoscopic Characterisation of Individual Endogenous Protein Aggregates in Human Neuronal Cells. *Chembiochem* **19**, 2033–2038 (2018).

5. Edelstein, A. D. *et al.* Advanced methods of microscope control using μManager software. *J. Biol. Methods* **1**, 10 (2014).

6. vonDiezmann, A., Shechtman, Y. &Moerner, W. E. Three-Dimensional Localization of Single Molecules for Super-Resolution Imaging and Single-Particle Tracking. *Chem. Rev.* **117**, 7244–7275 (2017).

7. Schindelin, J. *et al.* Fiji: an open-source platform for biological-image analysis. *Nat. Methods* **9**, 676–682 (2012).

8. Fröhlich, E. *et al.* Comparison of two in vitro systems to assess cellular effects of nanoparticles-containing aerosols. *Toxicol. Vitr.* **27**, 409–417 (2013).

9. Courte, J. *et al.* The expression level of alpha-synuclein in different neuronal populations is the primary determinant of its prion-like seeding. *Sci. Rep.* **10**, 4895 (2020).

Code name	Sequence (5’-3’)
**IS1**	CCAGATGTAT-CY3B
**Thiol-DS1**	Thiohexyl-TTATACATCTA
**DBCO-DS1**	DBCO-TTATACATCTATTTTTTTTTTTTTTTTTTTT

Table S1. Sequences of synthetic oligonucleotides. The complementary nucleotides that form a transient 8bp duplex during antibody-DNA PAINT imaging are underlined. DBCO = dibenzocyclooctyne tetraethylene glycol.

**Parameter**	Mean	SD	Max likelihood value	Cl1 lower	Cl1 upper	CI2 lower	CI2 upper
**Plateau**	60.57	7.91	58.57	51.43	68.57	45.71	77.14
**Initial fraction F_0**	-1.17	0.11	-1.14	-1.28	-1.07	-1.39	-0.96
**Rate k**	0.25	0.07	0.22	0.17	0.32	0.14	0.41

Table S2. Bayesian analysis for the replication of intracellular α-syn aggregates. The statistics show the fit results as means, maxima, standard deviations (SD) and credible intervals (CI). CI is presented as the 68% CI (CI1) and the 95% CI (CI2), corresponding to one and two standard deviations respectively.
