# Supplementary material for: Super-resolution imaging reveals α-synuclein seeded aggregation in SH-SY5Y cells
Source: Commun Biol. 2021 May 21;4:613. doi: 10.1038/s42003-021-02126-w (PMC8139990; doi:10.1038/s42003-021-02126-w)
Supplement: Supplementary file 3 — Description of Additional Supplementary Files [file 42003_2021_2126_MOESM3_ESM.pdf]

Description of Additional Supplementary Files

File name: Supplemental Movie 1
Description: Seeded24h cells imaged with AD-PAINT (1x magnification). The brightest spots with constant intensity are fiducial markers.

File name: Supplemental Movie 2
Description: Seeded24h cells imaged with AD-PAINT (10x magnification).

File name: Supplemental Movie 3
Description: PFFs imaged with AD-PAINT (1x magnification). The brightest spots with constant intensity are fiducial markers.

File name: Supplemental Movie 4
Description: PFFs imaged with AD-PAINT (10x magnification).

File name: Supplemental Data 1
Description: Source data for the main figures.
Sheet 1: original dataset of triplicated experiments for fig. 1c-f, with bold headings
Sheet 2: original dataset of triplicated experiments for fig. 2c-f, with bold headings
Sheet 3: original dataset of triplicated experiments for fig. 3b-d, with bold headings
Sheet 4: original dataset of triplicated experiments for fig. 4a-c, with bold headings
