# Supplementary material for: Super-resolution imaging reveals α-synuclein seeded aggregation in SH-SY5Y cells
Source: Commun Biol. 2021 May 21;4:613. doi: 10.1038/s42003-021-02126-w (PMC8139990; doi:10.1038/s42003-021-02126-w)
Supplement: Supplementary file 9 — Reporting Summary [file 42003_2021_2126_MOESM9_ESM.pdf]

nature research

Corresponding author(s): David Klenerman

Last updated by author(s): Jun 12, 2020

Reporting Summary

Nature Research wishes to improve the reproducibility of the work that we publish. This form provides structure for consistency and transparency in reporting. For further information on Nature Research policies, see our Editorial Policies and the Editorial Policy Checklist.

Statistics

For all statistical analyses, confirm that the following items are present in the figure legend, table legend, main text, or Methods section.

n/a	Confirmed	
[ ]	[x]	The exact sample size (*n*) for each experimental group/condition, given as a discrete number and unit of measurement
[ ]	[x]	A statement on whether measurements were taken from distinct samples or whether the same sample was measured repeatedly
[ ]	[x]	The statistical test(s) used AND whether they are one- or two-sided<br>*Only common tests should be described solely by name; describe more complex techniques in the Methods section.*
[x]	[ ]	A description of all covariates tested
[ ]	[x]	A description of any assumptions or corrections, such as tests of normality and adjustment for multiple comparisons
[ ]	[x]	A full description of the statistical parameters including central tendency (e.g. means) or other basic estimates (e.g. regression coefficient) AND variation (e.g. standard deviation) or associated estimates of uncertainty (e.g. confidence intervals)
[ ]	[x]	For null hypothesis testing, the test statistic (e.g. *F*, *t*, *r*) with confidence intervals, effect sizes, degrees of freedom and *P* value noted<br>*Give P values as exact values whenever suitable.*
[x]	[ ]	For Bayesian analysis, information on the choice of priors and Markov chain Monte Carlo settings
[x]	[ ]	For hierarchical and complex designs, identification of the appropriate level for tests and full reporting of outcomes
[x]	[ ]	Estimates of effect sizes (e.g. Cohen's *d*, Pearson's *r*), indicating how they were calculated

Our web collection on statistics for biologists contains articles on many of the points above.

Software and code

Policy information about availability of computer code

Data collection	Images acquired using open source Micro-Manager 1.4.
Data analysis	Image analysis was performed using custom scripts programmed in Jython 2.7 and Python 3.5.

For manuscripts utilizing custom algorithms or software that are central to the research but not yet described in published literature, software must be made available to editors and reviewers. We strongly encourage code deposition in a community repository (e.g. GitHub). See the Nature Research guidelines for submitting code & software for further information.

Data

Policy information about availability of data

All manuscripts must include a data availability statement. This statement should provide the following information, where applicable:

- Accession codes, unique identifiers, or web links for publicly available datasets
- A list of figures that have associated raw data
- A description of any restrictions on data availability

The data that support the findings of this study are available from the corresponding author upon reasonable request.

Field-specific reporting

Life sciences study design

All studies must disclose on these points even when the disclosure is negative.

Sample size	No formal power calculations were performed. Sample sizes were determined to be sufficient based on previous cell work of this type, the effect size, and consistency of the data generated throughout the course in this study.
Data exclusions	There were no data exclusions in this study.
Replication	The replicate experiments were successful. This forms the basis of the sample sizes described in the figure legends of each experiment.
Randomization	Not applicable in the cell culture-based experiments.
Blinding	Investigators were not blinded to samples, as the dependent measures were objective in nature.

Reporting for specific materials, systems and methods

We require information from authors about some types of materials, experimental systems and methods used in many studies. Here, indicate whether each material, system or method listed is relevant to your study. If you are not sure if a list item applies to your research, read the appropriate section before selecting a response.

Materials & experimental systems

n/a	Involved in the study	
☐	☒	Antibodies
☐	☒	Eukaryotic cell lines
☒	☐	Palaeontology and archaeology
☒	☐	Animals and other organisms
☒	☐	Human research participants
☒	☐	Clinical data
☒	☐	Dual use research of concern

Methods

n/a	Involved in the study	
☒	☐	ChIP-seq
☒	☐	Flow cytometry
☒	☐	MRI-based neuroimaging

Antibodies

Antibodies used	The following primary antibody was used: 1) Anti-Alpha-synuclein filament MJFR-14-6-4-2, BSA and Azide free (Abcam Cat. # ab214033); 2) 6E10 (Abcam Cat. # ab80627); 3) Syn211 (Biolegend Cat. # 803004); 4) IgG1 control antibody (clone COLIS69A; Kingfisher Biotech, Cat. # WS0799M-100) . The MJFR-14-6-4-2 antibody is manually labeled with a docking single-stranded DNA as described in Methods; 1:10,000 dilution for immunostaining. 6E10, Syn211, and IgG1 control antibody were diluted to 25 µg/mL for immunodepletion.
Validation	As per the manufacturer's website, MJFR-14-6-4-2 reacts with mouse, rat, and human alpha-synuclein and is suitable for dot blot, ICC/IF, IHC-P, and IHC-FoFr. Our results show at the dilution level used, no aggregates are detected from either healthy non-alpha-synuclein-seeded SH-SY5Y cells, or from monomeric alpha-synuclein-seeded cells (see Extended Data Fig. 3). 6E10, Syn211, and IgG1 control antibody are mouse anti-human antibodies according to the manufacturers' websites. The concentrations used for immunodepletion were optimized by validating with recombinant proteins in AD-PAINT imaging.

Eukaryotic cell lines

Policy information about cell lines

Cell line source(s)	The SH-SY5Y cells were obtained from Dr. Yu Ye.
Authentication	No additional authentication of this cell line was performed.
Mycoplasma contamination	Cells were tested negative for mycoplasma contamination.
Commonly misidentified lines (See ICLAC register)	No commonly misidentified cell lines were used.
